# The radiologist’s dilemma: Differentiating non-ischemic pneumatosis intestinalis from ischemic bowel necrosis

**DOI:** 10.1016/j.radcr.2026.09.018

**Published:** 2026-09-29

**Authors:** Ajay Khadka, Sujit Pant, Saurav Karki, Pratik Regmi, Saroj Raj K․C․

**Affiliations:** Department of Radiology, Shree Birendra Hospital (NAIHS), Chhauni, Nepal

**Keywords:** Pneumatosis intestinalis, Bowel ischemia, Intramural gas, Computed tomography

## Abstract

Pneumatosis intestinalis (PI) is defined by gas accumulation within the gastrointestinal wall and is frequently perceived as an indication of life-threatening bowel ischemia necessitating surgical intervention. However, PI can manifest as a benign entity, generating a significant diagnostic challenge between surgical and conservative management strategies. A 78-year-old male with chronic obstructive pulmonary disease (COPD) presented with mild abdominal pain and extensive small bowel intramural gas identified on computed tomography (CT), devoid of hemodynamic instability or lactic acidosis. Conservative management through bowel rest yielded substantial resolution of the intramural gas, successfully precluding surgery. While linear gas configurations frequently correlate with bowel ischemia, cystic morphologies – when assessed in conjunction with preserved bowel wall enhancement, absence of portal venous gas, and a stable clinical examination – should guide clinicians toward suspecting non-ischemic PI. Integrating CT imaging findings with clinical and biochemical parameters remains essential to circumventing unnecessary surgical exploration in non-ischemic presentations.

## Introduction

Pneumatosis intestinalis (PI) denotes the presence of gas within the wall of the gastrointestinal tract and has historically been interpreted as a hallmark of bowel ischemia, serving as a definitive indication for urgent exploratory laparotomy [1,2]. Contemporary multi-detector computed tomography (MDCT) has increased the frequency with which radiologists encounter incidental PI [3,4]. Misinterpreting this radiographic finding can result in high-risk, unnecessary surgical procedures or, conversely, a failure to treat necrotic bowel. Consequently, correlating gas morphology and ancillary radiological signs with the patient's overall clinical status is clinically paramount [5,6].

This manuscript details a case of non-surgical pneumatosis intestinalis to underscore the clinical importance of correlating imaging observations with patient presentation, with the objective of preserving diagnostic accuracy while avoiding unwarranted surgical interventions [7,8].

## Case report

A 78-year-old male presented to the emergency department complaining of mild abdominal pain. His medical history was notable for chronic obstructive pulmonary disease (COPD), with no documented history of recent abdominal surgery or trauma. Physical examination demonstrated a soft, non-tender abdomen with normal bowel sounds and a complete absence of peritonism or abdominal rigidity. Vital signs were stable, and the patient was afebrile.

Laboratory evaluations revealed a white blood cell count of 10,690/µL (reference range: 4–11 × 103/µL) and a serum lactate level of 1.2 mmol/L (reference range: 0.5–2.0 mmol/L), remaining well within normal limits. Comprehensive metabolic panels, inflammatory parameters, C-reactive protein (CRP), renal function profiles, and arterial blood gas assessments revealed no acute abnormalities, offering no biochemical evidence of systemic distress or bowel ischemia.

Contrast-enhanced computed tomography (CT) of the abdomen and pelvis was acquired in the portal venous phase using a multi-detector CT scanner (slice thickness: 1.25 mm; standard IV contrast dose administered). Initial evaluation utilizing limited abdominal radiography demonstrated mildly dilated bowel loops accompanied by clustered round air pockets within the bowel wall (Fig. 1A). The subsequent contrast-enhanced CT scan demonstrated extensive pneumatosis intestinalis involving the small bowel loops (Fig. 1B). The intramural gas exhibited a prominent cystic morphology localized predominantly to the submucosal layer. Crucially, there was no radiographic evidence of portal venous gas, mesenteric venous air, or free fluid. The bowel wall displayed normal contrast enhancement without pathological thickening, and the mesenteric vessels remained patent without signs of vascular occlusion or stranding (Fig. 1C).

**Fig. 1:**
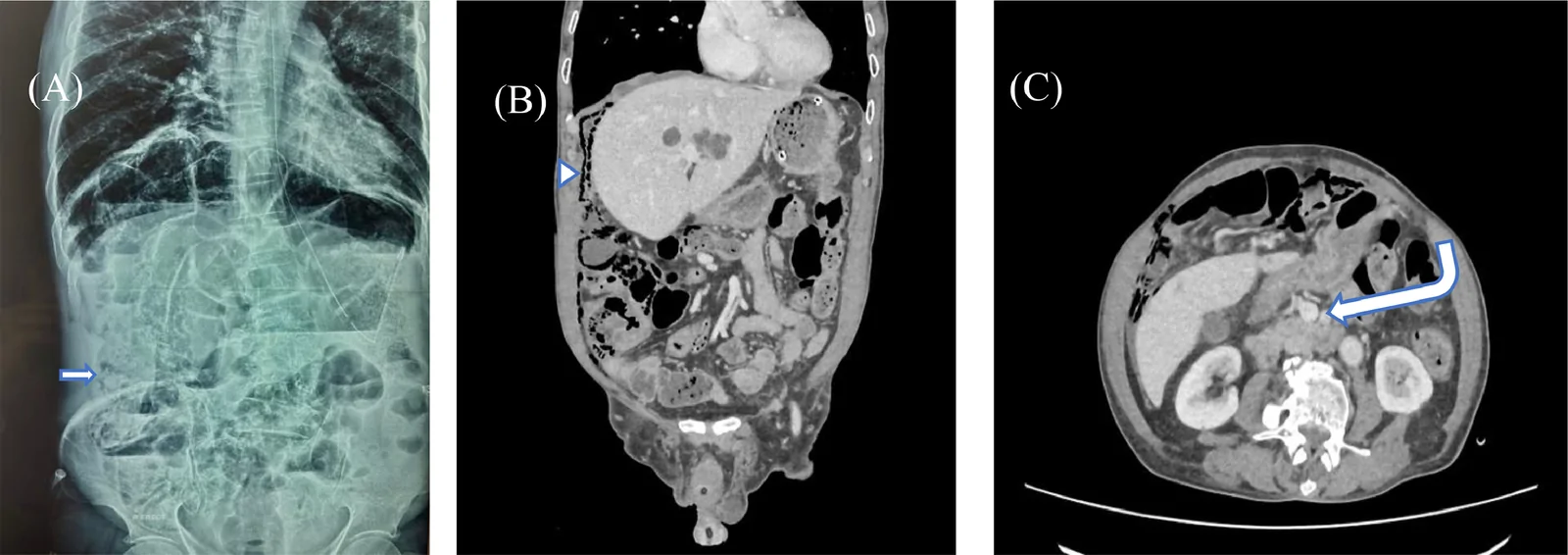
(A) Limited erect abdominal radiograph showing clusters of small rounded air pockets within the bowel wall – long arrow, (B) Representative coronal contrast-enhancement CT image in the portal venous phase showing extensive pneumatosis intestinalis in ileo-jejunal loops with preserved wall enhancement and patent mesenteric vasculature – arrow head. (C) Representative axial CT shows opacified inferior mesenteric artery – curved arrow.

The discrepancy between the imaging observation and the patient's clinical stability established a profound diagnostic challenge. Based on the comprehensive assessment of preserved enhancement, patent vessels, and the absence of warning signs on both CT imaging and laboratory assays, a diagnosis of non-ischemic pneumatosis intestinalis was favored.

Following multidisciplinary evaluation, formal surgical consultation was obtained and recommended non-operative management. The patient was admitted for close clinical observation and treated conservatively via bowel rest, intravenous fluid hydration, supplemental oxygen, and empiric supportive care. Serial abdominal examinations, white blood cell counts, and repeat serum lactate measurements remained consistently unremarkable throughout the hospital admission. Follow-up CT imaging performed on hospital day 4 using identical slice parameters and soft-tissue window setting demonstrated a significant near-complete reduction in the volume of intramural gas within the exact same bowel segment (Fig. 2). The patient was successfully advanced to a regular diet without symptom recurrence and was subsequently discharged in stable condition.

**Fig. 2:**
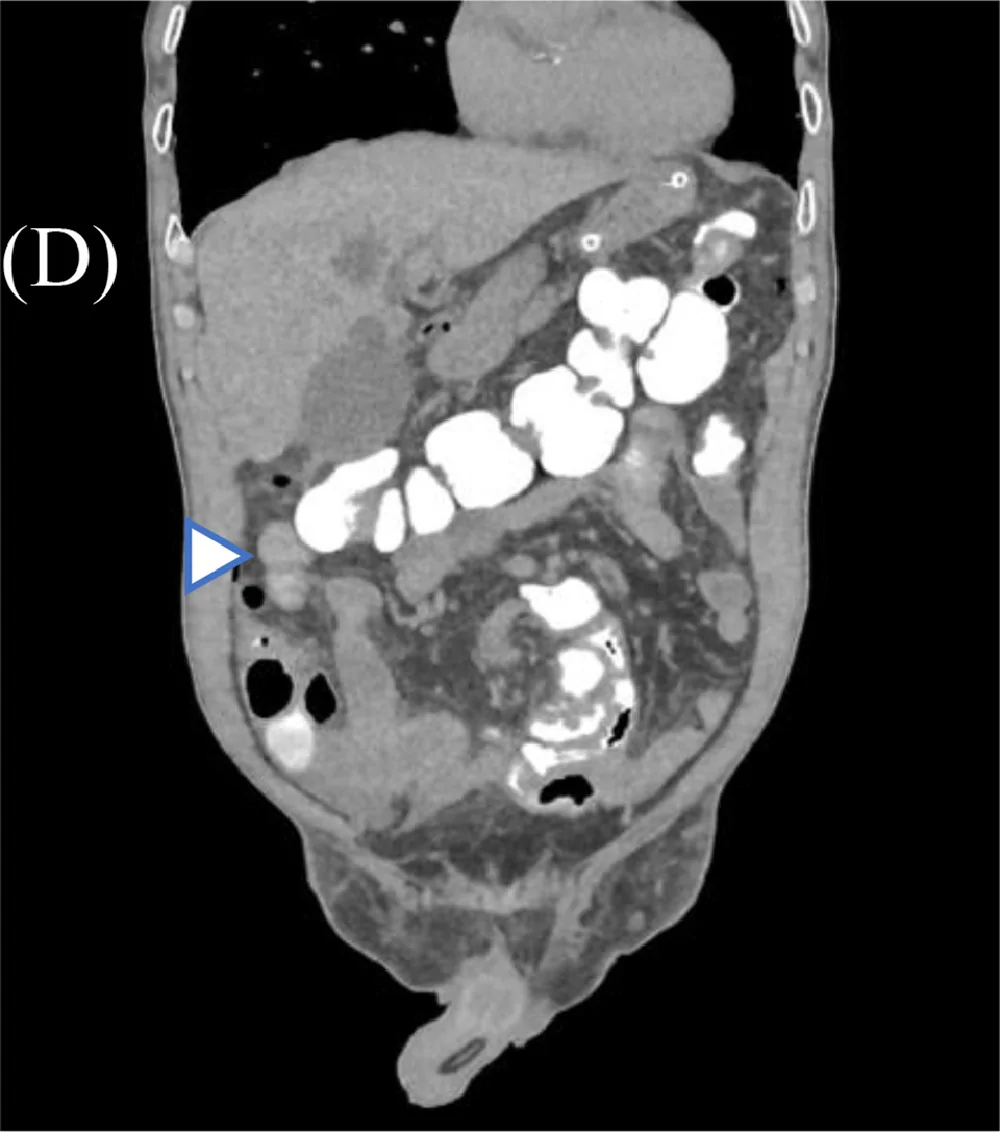
Coronal view abdominal computed tomography (CT) follow-up image (Day 4) utilizing identical soft-tissue window setting and anatomical planes relative to Figure 1B, showing near-complete interval resolution of pneumatosis intestinalis – arrow head.

## Discussion

Recognizing pneumatosis intestinalis remains a major diagnostic challenge. Although early surgical literature associated PT with high mortality rates, modern MDCT data indicates that approximately half of all identified cases are benign and do not require operative management [1,3,9].

Current literature establishes three primary pathophysiological theories explaining intramural gas in non-surgical patients [10]. The mechanical theory proposes that elevated intramural pressure—potentially associated with background conditions such as COPD-induced coughing - forces air across mucosal disruptions [4,10]. The pulmonary theory postulates that alveolar rupture causes pneumomediastinum, allowing gas to dissect along mesenteric vessels toward the bowel wall [4,10]. Finally, the bacterial theory suggests that gas-forming bacilli penetrate the submucosa, a mechanism often observed in immunocompromised hosts [4,5,10]. While COPD is noted in this patient’s history as a potential clinical association linked to mechanical mechanisms, a direct causative relationship could not be definitively established in the absence of documented acute pulmonary interstitial emphysema, pneumomediastinum, or recent severe coughing episodes.

Radiologically, gas morphology provides secondary diagnostic clues with cystic clusters frequently suggesting non-ischemic etiology while linear gas patterns prompts closer scrutiny for bowel infarction [4,6]. However, contemporary evidence underscores that the accompanying findings carry superior predictive value [4,7,9]. When ancillary signs such as bowel wall enhancement, wall thickening, portal venous gas, mesenteric stranding, free fluid, or occluded mesenteric vessels are absent, the probability of ischemia decreases substantially [3,4,7].

The clinical management decision relies on the integration of these imaging parameters with clinical stability. In the absence of red-flag indicators—such as metabolic acidosis, elevated serum lactate, peritoneal signs, or leukocytosis—patients can be managed safely through a non-operative strategy [[7], [8], [9]]. This case reinforces the clinical transition toward selective watchful waiting, confirming that isolated cystic PI in a stable clinical context represents a radiological sign rather than an absolute surgical mandate [4,6,8].

Limitations of this case report include the lack of histopathological confirmation due to conservative management and the absence of direct endoscopic mucosal inspection. However, serial physical evaluations and multi-parameter laboratory tracking effectively ensured patient safety, verifying the viability of a non-operative approach for carefully selected non-ischemic presentations.

## Conclusion

Pneumatosis intestinalis remains a classic diagnostic dilemma, spanning a clinical spectrum from incidental non-ischemic findings to catastrophic bowel ischemia. Intramural air is a radiological finding that must never be interpreted in isolation. While the presence of PI often triggers surgical consultation, a nuanced evaluation is essential to prevent the morbidity associated with unnecessary laparotomies. Successful navigation of this dilemma relies on an integrated assessment:

• **Radiological characterization:** Identifying gas morphology alongside the critical absence of adverse ancillary signs such as portal venous gas, abnormal wall enhancement, bowel wall thickening, or occluded mesenteric vessels.
• **Clinical and biochemical integration:** Acknowledging that a stable physical examination, normal serial leukocyte counts, and normal baseline serum lactate serve as vital components of a multi-parameter decision matrix supporting a non-operative strategy.

Evaluating the patient's clinical status alongside comprehensive intramural imaging ensures safer management and prevents the risks of unneeded surgical intervention.

## Patient consent

Written informed consent for publication of this case report and the accompanying radiological images was obtained from the patient. A copy of the written consent is available for review by the Editor-in-Chief of this journal upon request.
